# 3-deazaneplanocin A protects against cisplatin-induced renal tubular cell apoptosis and acute kidney injury by restoration of E-cadherin expression

**DOI:** 10.1038/s41419-019-1589-y

**Published:** 2019-05-01

**Authors:** Jun Ni, Xiying Hou, Xueqiao Wang, Yinfeng Shi, Liuqing Xu, Xiaoqing Zheng, Na Liu, Andong Qiu, Shougang Zhuang

**Affiliations:** 1 Department of Nephrology, Shanghai East Hospital, Tongji University School of Medicine, Shanghai, China; 2 Shanghai Institute of Immunology, Department of Immunology and Microbiology, Shanghai Jiao Tong University School of Medicine, Shanghai, China; 3 School of Life Sciences and Technology, Tongji University, Shanghai, China; 4 Department of Medicine, Rhode Island Hospital and Brown University School of Medicine, Providence, RI USA

**Keywords:** Pharmacology, Translational research

## Abstract

3-deazaneplanocin A (3-DZNeP) has been used as an inhibitor of enhancer of zeste homolog 2 (EZH2). Here, we explore the role and underlying mechanisms action of 3-DZNeP in abrogating cisplatin nephrotoxicity. Exposure of cultured mouse renal proximal tubular epithelial cells (mTECs) to cisplatin resulted in dose and time-dependent cleavage of caspase-3, decrease of cell viability, and increase of histone H3 lysine 27 trimethylation (H3K27me3), whereas expression levels of EZH2, a major methyltransferase of H3K27me3, were not affected. Treatment with 3-DZNeP significantly inhibited cisplatin-induced activation of caspase-3, apoptosis, loss of cell viability but did not alter levels of EZH2 and H3K27me3 in cultured mTECs. 3-DZNeP treatment did not affect activation of extracellular signal-regulated kinase (ERK) 1/2, p38 or c-Jun N-terminal kinases (JNK) 1/2, which contribute to renal epithelial cell death, but caused dose-dependent restoration of E-cadherin in mTECs exposed to cisplatin. Silencing of E-cadherin expression by siRNA abolished the cytoprotective effects of 3-DZNeP. In contrast, 3-DZNeP treatment potentiated the cytotoxic effect of cisplatin in H1299, a non-small cell lung cancer cell line that expresses lower E-cadherin levels. Finally, administration of 3-DZNeP attenuated renal dysfunction, morphological damage, and renal tubular cell death, which was accompanied by E-cadherin preservation, in a mouse model of cisplatin nephrotoxicity. Overall, these data indicate that 3-DZNeP suppresses cisplatin-induced tubular epithelial cell apoptosis and acute kidney injury via an E-cadherin-dependent mechanism, and suggest that combined application of 3-DZNeP with cisplatin would be a novel chemotherapeutic strategy that enhances the anti-tumor effect of cisplatin and reduces its nephrotoxicity.

## Introduction

Acute kidney injury (AKI) characterized by abrupt deterioration in kidney function and tubular cell death is associated with high morbidity and mortality^1^. It can be caused by multiple pathological conditions, such as ischemia-reperfusion (I/R), sepsis, trauma, and nephrotoxic agents, including drugs with therapeutic uses^2,3^. Nephrotoxic AKI constitute approximately one-third of patients with AKI^3^. Among the nephrotoxic agents that induce AKI, cisplatin (dichlorodiamino platinum), a chemotherapeutic drug that has been extensively used in chemotherapy, is most investigated in vitro and in vivo models of AKI. Although cisplatin has a significant antitumor effect in various solid tumors such as non-small cell lung cancer (NSCLC) and prostate cancer^4^, its clinical application is limited by its various side effects^5–8^ with nephrotoxicity, one of cisplatin’s most common side effects^9^. Approximately one-third of patient undergoing cisplatin treatment suffers from this disorder, and there is no effective therapeutic strategy to protect against its nephrotoxicity currently^6,10^. Finding agents that can ameliorate cisplatin-induced AKI is a critical challenge given its widespread use as chemotherapy.

The cellular and molecular mechanisms by which cisplatin induces AKI have been looked at extensively. Cisplatin is taken up through the organic cation transporters 2 located on the basolateral side of tubular cells^11,12^, and its accumulation can result in both apoptosis and necrosis of renal tubular cells^13^. Apoptosis is a type of programed cell death that is predominantly mediated by the caspase pathway. Caspase-3 plays a primary role, and its cleavage represents its activation. Other cellular events involved in apoptosis include mitochondrial damage and activation of mitogen-activated protein kinases (MAPK), including extracellular signal-regulated kinase 1/2 (ERK1/2), p38, and c-Jun N-terminal kinases (JNK)^14–17^. In addition, disruption of epithelial cell integrity by inhibition or downregulation of cellular adhesion molecules such as E-cadherin also promotes renal tubular cell apoptosis^18^.

Recently, our studies showed that ischemia/reperfusion injury to the kidney or oxidant injury to the cultured proximal tubular cells, resulted in activation of enhancer of zeste homolog 2 (EZH2), a methyltransferase that induces histone H3 lysine 27 trimethylation (H3K27me3), a well-known repressive marker, and induced renal epithelial cell death. This was evidenced by our observations that inhibition of EZH2 by 3-deazaneplanocin A (3-DZNeP) attenuated AKI or/and renal tubular cell death and restored E-cadherin expression^19^. 3-DZNeP is an inhibitor of S-adenosyl-l-homocysteine hydrolase (SAHH), which is known to inhibit EZH2. Pharmacologically, 3-DZNeP can promote degradation of EZH2^20^ and subsequently reduce H3K27 me3 levels^21^. EZH2 has been shown to be overexpressed in many aggressive tumors^22–24^, and H3K27me3 is responsible for the repression and heterochromatin formation of various tumor suppressor genes^25,26^. Pharmacological inhibition of EZH2 has been reported to be effective in animal models in the treatment of multiple cancers, such as myeloma^27^, leukemia^28^, lymphoma^29^, gastric cancer^30^, chondrosarcoma^31^, and lung cancer, especially NSCLC^32,33^. Moreover, 3-DZNeP increased sensitivity of lung adenocarcinoma cells to cisplatin treatment^34^.

Since application of 3-DZNeP can attenuate kidney cell apoptosis and tissue damage in the murine model of ischemia/reperfusion-induced AKI and enhance cisplatin-induced cell death in cancer cells, we investigated whether 3-DZNeP would be able to protect kidneys from cisplatin-induced nephrotoxicity and to potentiate its chemotherapeutic effects in cancer cells. Our results demonstrated that 3-DZNeP protects against cisplatin-induced tubular cell injury in cultured mouse renal proximal tubular epithelial cells (mTECs) and in a mouse model of cisplatin nephrotoxicity and enhances the cytotoxic effect of cisplatin in tumor cells (i.e. NSCLC cells) through a mechanism involving the upregulation of E-cadherin expression. This finding suggests that the combination of 3-DZNeP and cisplatin as treatment of various tumors may increase the efficacy of cisplatin in treating cancer while protecting the kidneys from cisplatin-induced tubular damage.

## Results

### Cisplatin-induced apoptosis of renal tubular cells is accompanied by increased levels of H3K27me3, but not EZH2

**Fig. 1:**
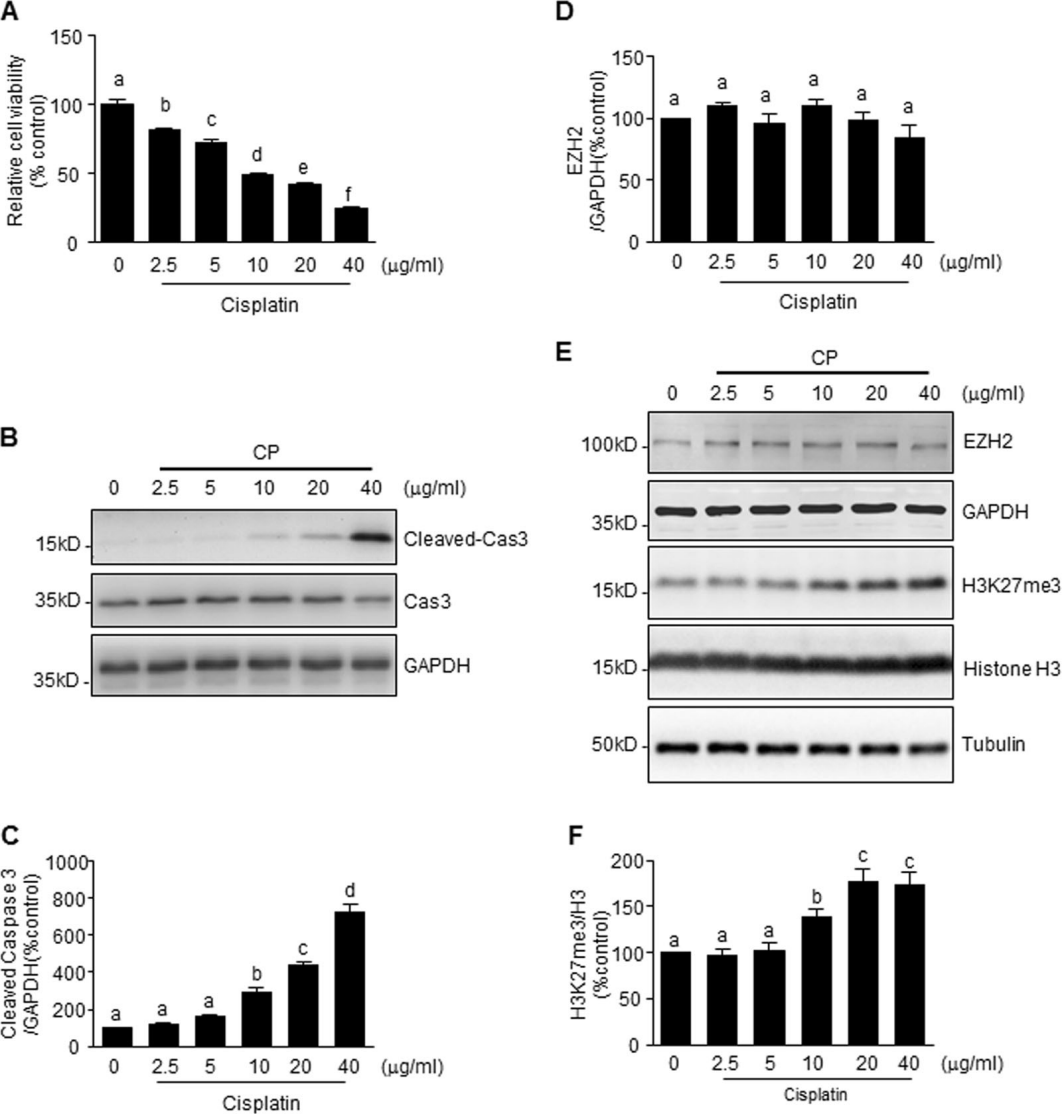
Cisplatin (CP) induces mouse renal proximal tubular epithelial cell (mTEC) apoptosis and H3 lysine 27 trimethylation (H3K27me3) in a dose-dependent manner. mTECs were exposed to various concentrations of cisplatin for 24 h. Cell viability was detected by cell counting kit-8 (CCK-8) assay (**a**). Cell lysates were prepared and subjected to immunoblot analysis with antibodies against caspase3 (cas3) and GAPDH (**b**), enhancer of zeste homolog 2 (EZH2), H3K27me3, GAPDH, H3, and tubulin (**e**). The levels of cleaved-cas3 (**c**), EZH2 (**d**), or H3K27me3 (**f**) were quantified by densitometry and normalized with GAPDH or histone H3 as indicated. Data represent the mean ± SEM of at least three experiments. Bars with different letters (**a**–**f**) for each molecule are significantly different from one another (*P* < 0.05)

**Fig. 2:**
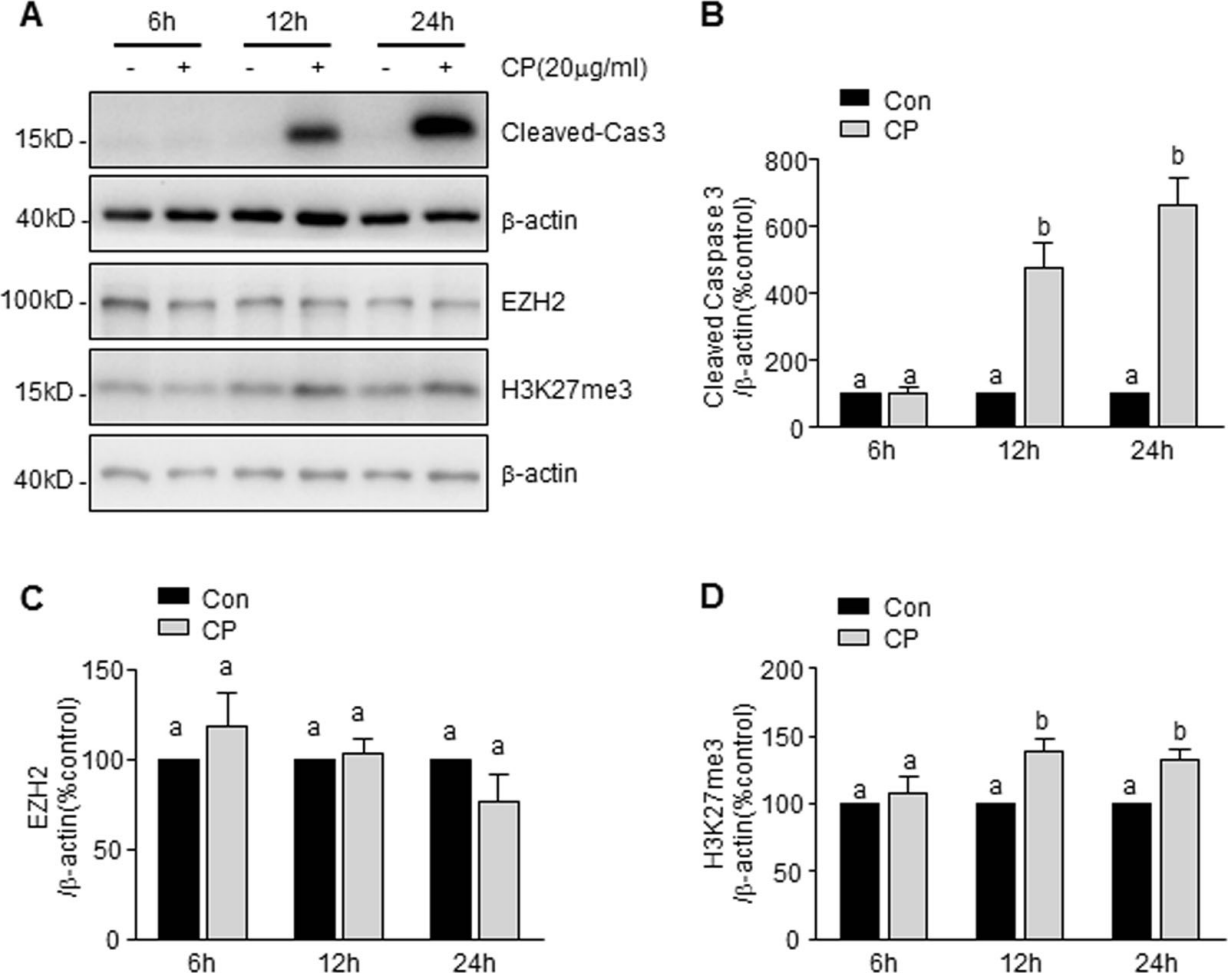
CP induces mTEC apoptosis and H3K27me3 in a time-dependent manner. mTECs were treated with 20 μg/ml of cisplatin for the indicated time. Cell lysates were prepared and subjected to immunoblot analysis with antibodies against Cleaved caspase 3(Cleaved-Cas3), EZH2, H3K27me3, and β-actin (**a**). The levels of cleaved-cas3 (**b**), EZH2 (**c**), or H3K27me3 (**d**) were quantified by densitometry and normalized with β-actin. Data represent the mean ± SEM of at least three experiments. Bars with different letters (**a**–**c**) for each molecule are significantly different from one another (*P* < 0.05)

Our recent study demonstrated that inhibition of EZH2 activity by 3-DZNeP protects against renal tubular cell injury in a murine model of I/R-induced AKI and cultured mouse renal proximal tubular cells. To understand whether this pharmacological action of 3-DZNeP is also implicated in cisplatin nephrotoxicity, we first examined EZH2 and H3K27me3 levels during the course of cisplatin-induced cell death in cultured mTECs. As shown in Fig. 1a, exposure of mTECs to cisplatin resulted in a loss of cell viability, as measured by the CCK-8 assay, which occurred in a dose-dependent manner, with a significant reduction at 2.5 μg/ml and maximum reduction at 40 μg/ml. Immunoblot analysis demonstrated that cisplatin treatment also induced a dose-dependent increase of cleaved-caspase3, with significant changes when cells were exposed to it from 10 to 40 μg/ml (Fig. 1b, c). The time course study with 20 μg/ml of cisplatin showed a significant increase of cleaved-caspase3 at 12 h and further increase at 24 h (Fig. 2a, b).

To investigate whether EZH2 expression and its methyltransferase activity are involved in the cytotoxicity of cisplatin, we next examined the effect of cisplatin on the expression of EZH2 and H3K27me3, the major form of methylated histone H3 lysine 27. Cisplatin increased the level of H3K27me3 in a dose-dependent (Fig. 1e, f) and time-dependent manner (Fig. 2a, d), but did not significantly affect the expression level of EZH2 (Figs. 1d, e and 2a, c). This suggests that cisplatin-induced H3K27me3 may be influenced by the elevated methyltransferase activity of EZH2 but not associated with its protein levels in mTECs.

### 3-DZNeP inhibits cisplatin-induced tubular cell apoptosis by an EZH2/H3K27me3 independent pathway

**Fig. 3:**
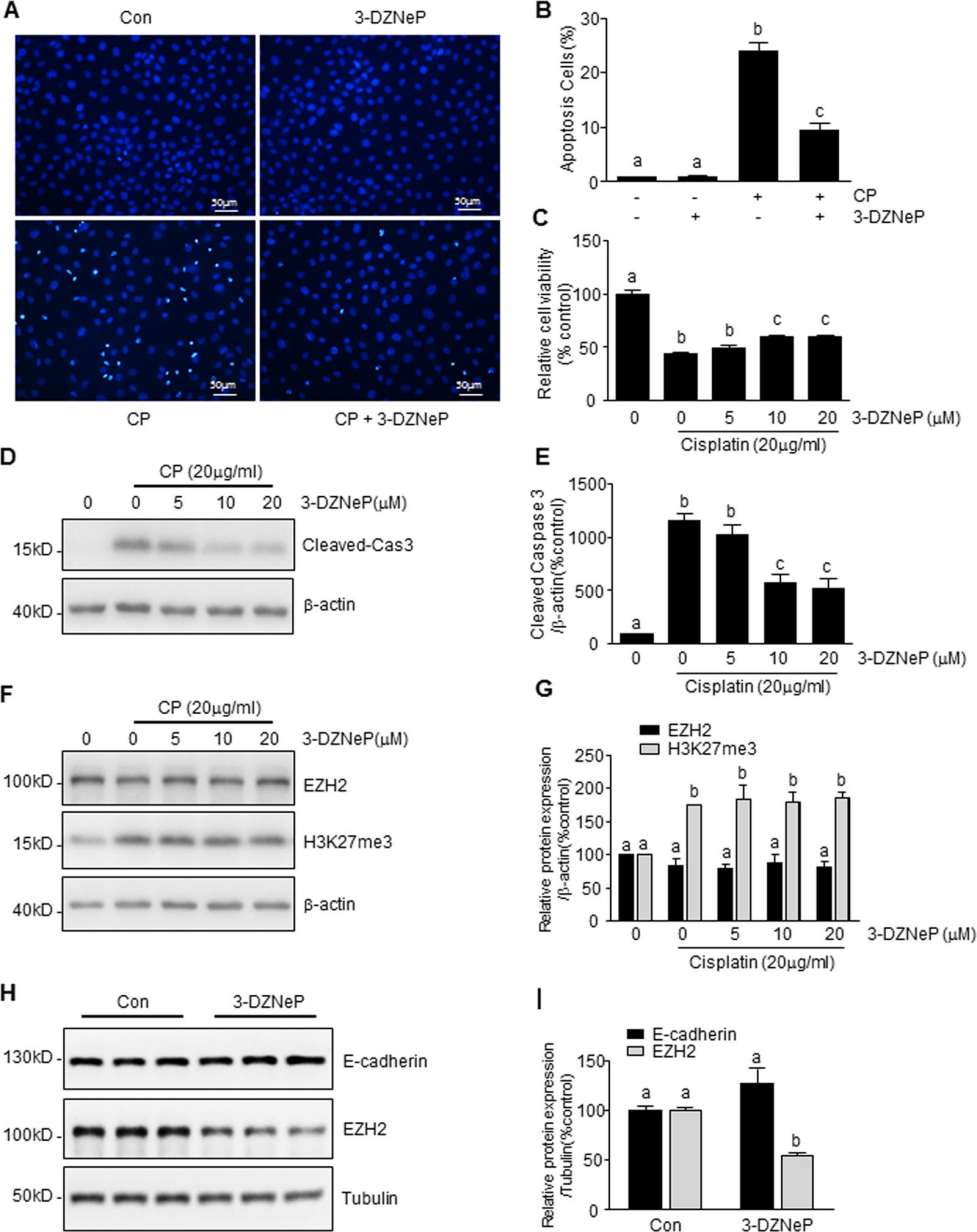
3-deazaneplanocin A (3-DZNeP) suppresses CP-induced mTEC apoptosis via an EZH2/H3K27me3 independent pathway. mTECs were pretreated with 3-DZNeP [10 μM (**a**, **b**)] or [0–10 μM (**c–g**) or for 30 min and then exposed to cisplatin (20 μg/ml) for an additional 24 h or treated with 3-DZNeP (10 μM) alone for 24 h (**h**, **i**). Cell apoptosis was detected by 4′,6-diamidino-2-phenylindole (DAPI) staining and the incidence of apoptosis was analyzed by enumerating nuclei of deep dying cells with condensed chromatin (**a**), and expressed as the percentage of apoptotic cells (**b**). Cell viability was detected by CCK-8 assay (**c**). Cell lysates were prepared and subjected to immunoblot analysis with antibodies against caspase3 (cas3), EZH2, H3K27me3, E-cadherin, tubulin, and β-actin (**d**, **f**, **h**). The levels of cleaved-caspase3 (**e**), EZH2 (**g**, **i**), H3K27me3 (**g**), or E-cadherin (**i**) were quantified by densitometry and normalized with tubulin or β-actin. Data represent the mean ± SEM of at least three experiments. Bars with different letters (**a**–**c**) for each molecule are significantly different from one another (*P* < 0.05)

3-DZNeP has been reported as an inhibitor of EZH2 with anti-tumor and renal protective effects^19,34^. To investigate the role of EZH2/H3K27me3 in renal tubular cell apoptosis, we examined the effect of 3-DZNeP on cisplatin-induced tubular cell apoptosis. Figure 3 shows that pretreatment with 3-DZNeP dose-dependently ameliorated apoptosis, as represented by nuclear staining by 4′,6-diamidino-2-phenylindole (DAPI) (Fig. 3a, b), inhibited cisplatin-induced loss of cell viability (Fig. 3c) and as well as by cisplatin-induced caspase3-cleavege (Fig. 3d, e). Since the morphological appearance of condensed nuclei and induction of caspase cleavage are hallmarks of apoptosis, these results confirmed that cisplatin is toxic to renal tubular cells and induces programmed cell death. Surprisingly, at the doses (10–20 μM), which inhibited cisplatin-induced apoptosis, 3-DZNeP did not affect levels of EZH2 and H3K27me3 in renal tubular epithelial cells exposed to cisplatin (Fig. 3f, g). Notably, 3-DZNeP at 10 μM is still able to reduce EZH2 protein levels in the same type of cells without cisplatin treatment, while E-cadherin expression was not affected by this agent (Fig. 3h, i). These data indicate that protection against apoptosis of renal epithelial cells by 3-DZNeP may not be through an EZH2 or H3K27me3-dependent mechanisms. The data further indicate that EZH2 is sensitive to degradation induced by 3-DZNeP in normal renal tubular epithelial cells, but resistant to 3-DZNeP in the same cell type exposed to cisplatin.

### Knockdown of EZH2 enhances cisplatin-induced tubular cell apoptosis

**Fig. 4:**
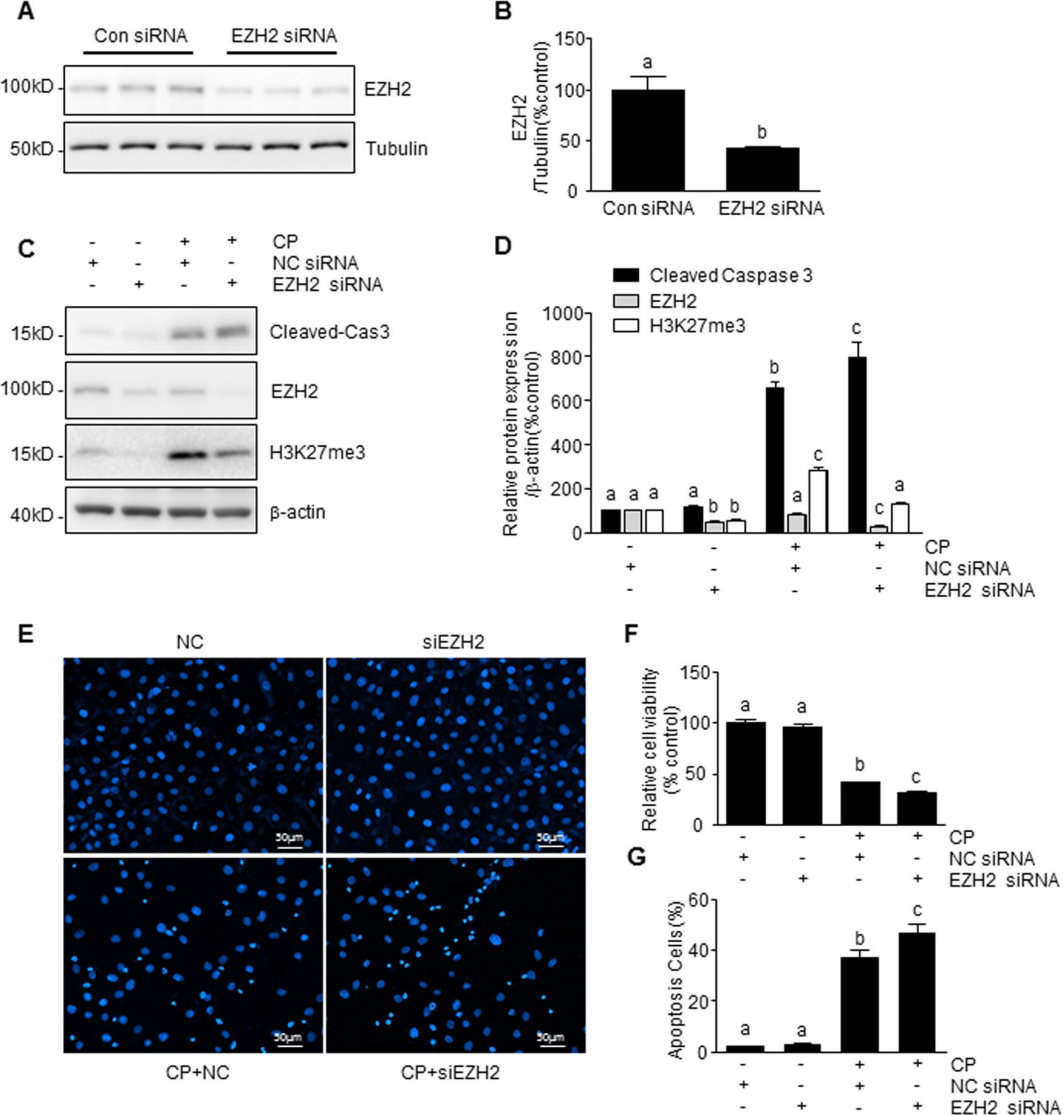
Knockdown of EZH2 enhanced CP-induced mTEC apoptosis. mTECs were transfected with siRNA targeting EZH2 or scrambled siRNA (**a–g**) and then exposed to cisplatin (20 μg/ml) for an additional 24 h (**c–g**). Cell lysates were prepared and subjected to immunoblot analysis with antibodies against cas3, EZH2, H3K27me3, tubulin, and β-actin (**a**, **c**). The levels of cleaved-cas3 (**d**), EZH2 (**b**, **d**), or H3K27me3 (**d**) were quantified by densitometry and normalized with tubulin or β-actin. Cell viability was detected by CCK-8 assay (**f**). Cell apoptosis was detected by DAPI staining and the incidence of apoptosis was analyzed by enumerating nuclear of deep dyeing cells with condensed chromatin (**e**), and expressed as the percentage of apoptotic cells (**g**). Data represent the mean ± SEM of at least three experiments. Bars with different letters (**a**–**c**) for each molecule are significantly different from one another (*P* < 0.05)

To validate the role of EZH2 in cisplatin-induced tubular cell apoptosis, we further examined the effect of EZH2 silencing on caspase 3-cleavege, cell viability, and chromatin condensation in mTECs. Transfection of tubular cells with specific small interfering RNA (siRNA) to EZH2 resulted in a significant down-regulation of EZH2 (Fig. 4a, b). The knockdown of EZH2 also significantly decreased H3K27me3 and enhanced expression of cisplatin-induced caspase 3 cleavage, loss of cell viability, and chromatin condensation compared with cells transfected with control siRNA (Fig. 4c–g). These results are opposite to the inhibitory effect of 3-DZNeP on renal tubular cell death, and suggest a protective effect for basal levels of EZH2 in renal tubular cells. They further suggest that 3-DZNeP-mediated renoprotection is not through EZH2 and/or H3K27 trimethylation in this cell type.

### 3-DZNeP treatment does not affect activation of ERK/1/2, p38, and JNK/1/2, but preserves E-cadherin protein levels in mTECs exposed to cisplatin

**Fig. 5:**
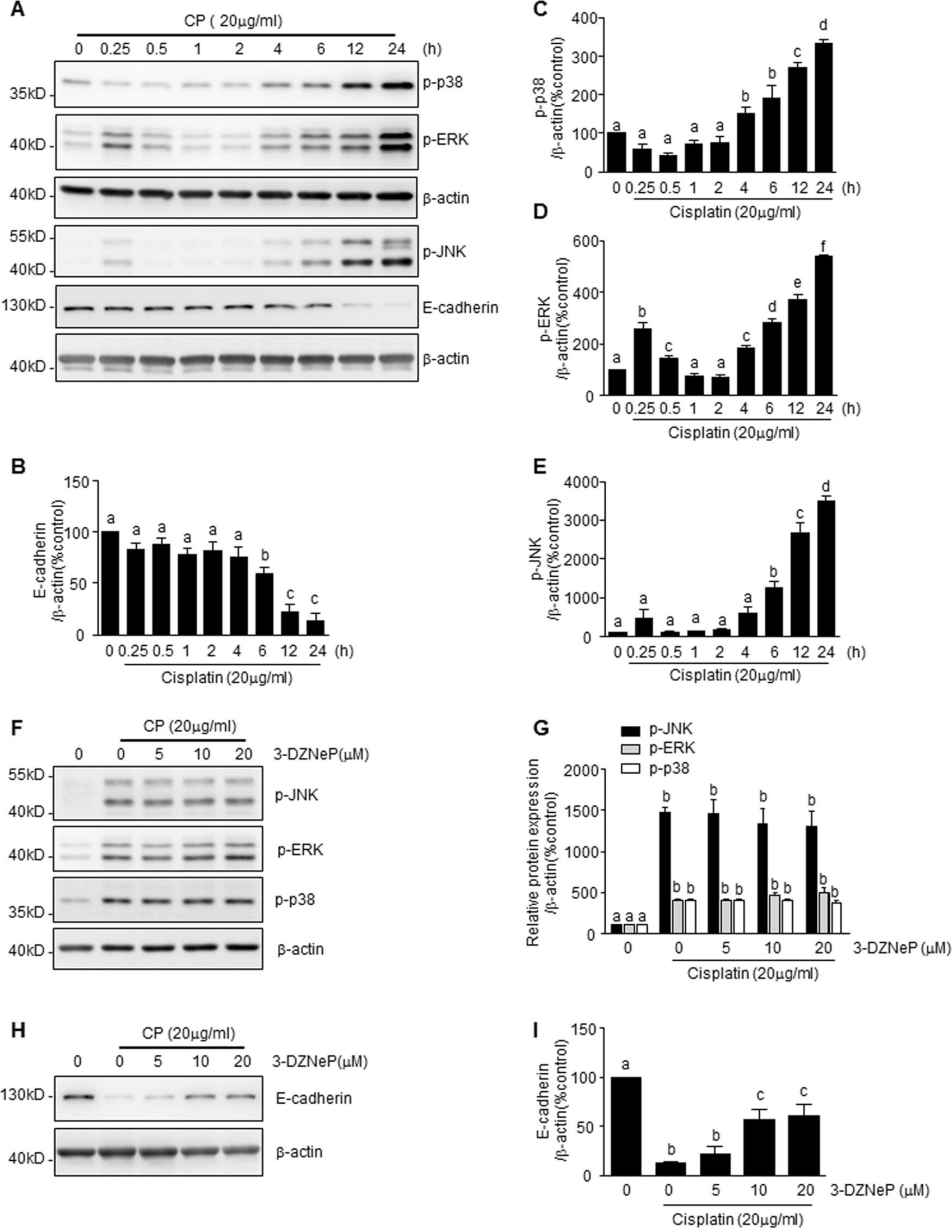
3-DZNeP inhibits CP-induced mTEC apoptosis by a mitogen-activated protein kinase (MAPK) independent restoration of E-cadherin. mTECs were treated with 20 μg/ml of cisplatin for 0–24 h (**a**–**e**) or pretreated with 3-DZNeP (0–20 μM) and then exposed to cisplatin (20 μg/ml) for an additional 24 h (**f**–**i**). Cell lysates were prepared and subjected to immunoblot analysis with antibodies against JNK phosphorylation (p-JNK), ERK phosphorylation (p-ERK), p38 phosphorylation (p-p38), E-cadherin, and β-actin (**a**, **f**, **h**). The levels of p-JNK (**e**, **g**), p-ERK (**d**, **g**), p-p38(**c**, **g**), or E-cadherin (**b**, **i**) were quantified by densitometry and normalized with β-actin. Data represent the mean ± SEM of at least three experiments. Bars with different letters (**a**–**f**) for each molecule are significantly different from one another (*P* < 0.05)

Activation of ERK, p38, or JNK, and loss of E-cadherin contribute to tubular cell apoptosis induced by cisplatin^17,19,35^. To examine MAPK activation and E-cadherin protein level in response to cisplatin treatment and the effect of 3-DZNeP, mTECs were exposed to cisplatin (20 μg/ml) for different periods of time (Fig. 5). Presence of cisplatin increased p38, ERK, and JNK phosphorylation (Fig. 5a, c–e) in a time-dependent manner, peaking at 24 h. Conversely, cisplatin time-dependently reduced E-cadherin protein levels, with the lowest level at 24 h (Fig. 5a, b). Treatment with 3-DZNeP did not, however, affect the phosphorylation of those kinases in cisplatin-treated mTECs (Fig. 5f, g). Interestingly, 3-DZNeP treatment dose-dependently restored E-cadherin protein levels, which had been suppressed by cisplatin (Fig. 5h, i). Taken together, it appears that 3-DZNeP protects against cisplatin nephrotoxicity through a mechanism associated with preservation of E-cadherin expression rather than inhibition of MAPK signaling pathways.

### 3-DZNeP enhances cisplatin-induced tubular cell apoptosis in mTECs with E-cadherin silencing

**Fig. 6:**
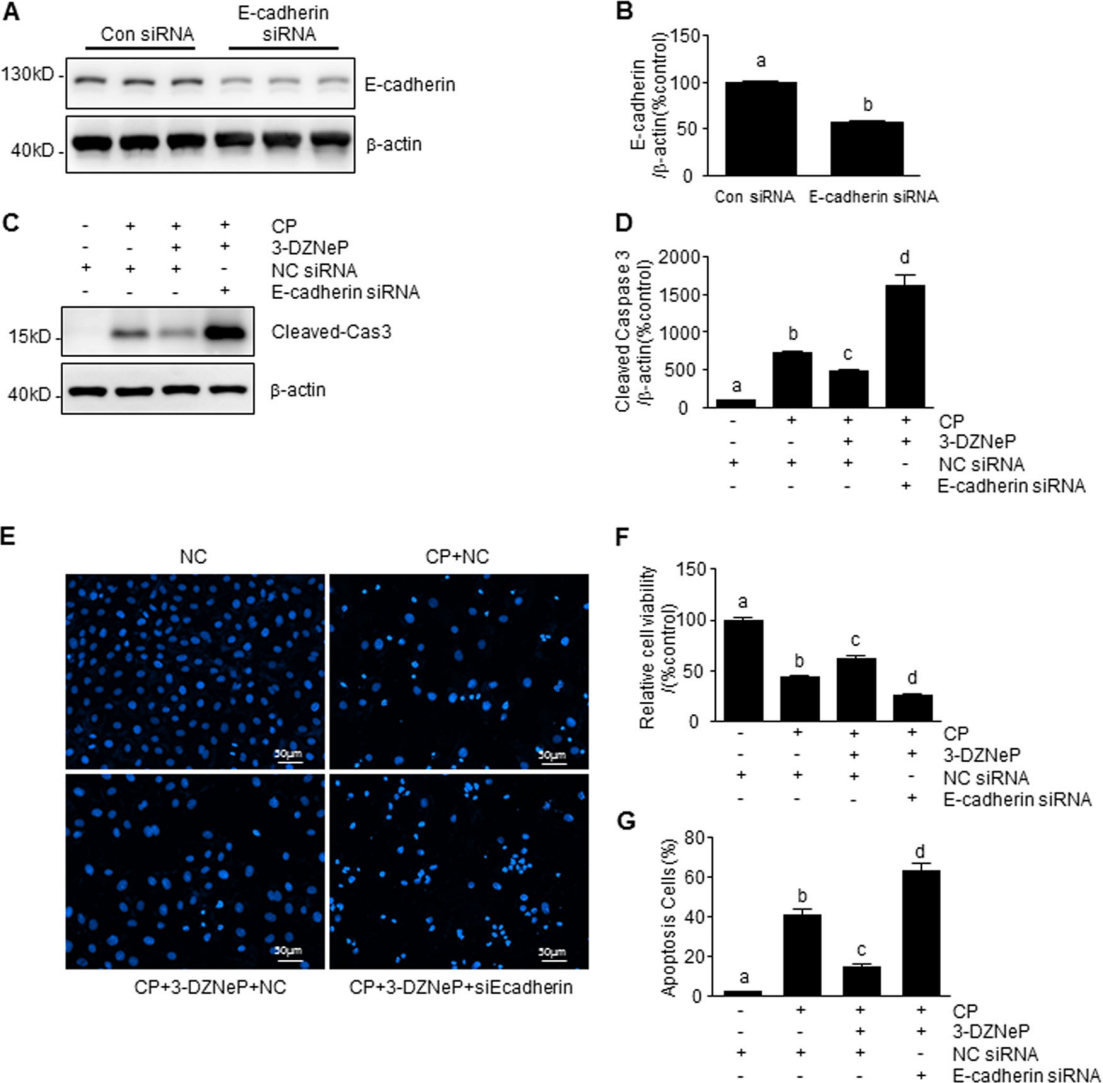
3-DZNeP enhanced CP-induced tubular cell apoptosis in E-cadherin silenced mTECs. mTECs were transfected with siRNA targeting E-cadherin or scrambled siRNA (**a–g**) and then treated with 3-DZNeP (10 μM) for 30 min and then exposed to cisplatin (20 μg/ml) for an additional 24 h (**c–g**). Cell lysates were prepared and subjected to immunoblot analysis with antibodies against E-cadherin, cas3, and β-actin (**a**, **c**). The levels of E-cadherin (**b**) or cleaved-cas3 (**d**) were quantified by densitometry and normalized with β-actin. Cell viability was detected by CCK-8 assay (**f**). Cell apoptosis was detected by DAPI staining (**e**) and the incidence of apoptosis was analyzed by enumerating nuclear of deep dyeing cells with condensed chromatin, and expressed as the percentage of apoptotic cells (**g**). Data represent the mean ± SEM of at least three experiments. Bars with different letters (**a**–**d**) for each molecule are significantly different from one another (*P* < 0.05)

To confirm the role of E-cadherin in the anti-apoptotic effect of 3-DZNeP in cisplatin-induced tubular cell apoptosis, we examined the effect of 3-DZNeP on cisplatin-induced caspase 3 cleavage, loss of cell viability, and chromatin condensation in E-cadherin-silenced mTECs. Transfection of tubular cells with siRNA specifically for E-cadherin significantly reduced E-cadherin protein levels (Fig. 6a, b) and subsequently diminished the anti-apoptotic effect of 3-DZNeP in renal tubular epithelial cells following cisplatin treatment, resulting in enhanced caspase 3 cleavage, loss of cell viability, and chromatin condensation compared with cells transfected with control siRNA (Fig. 6c–g). Therefore, preservation of E-cadherin levels is essential for 3-DZNeP to confer a renoprotective effect against cisplatin-induced renal tubular cell death.

### 3-DZNeP enhances cisplatin-induced apoptosis via the inhibition of EZH2/H3K27me3 pathway in NSCLC cells

**Fig. 7:**
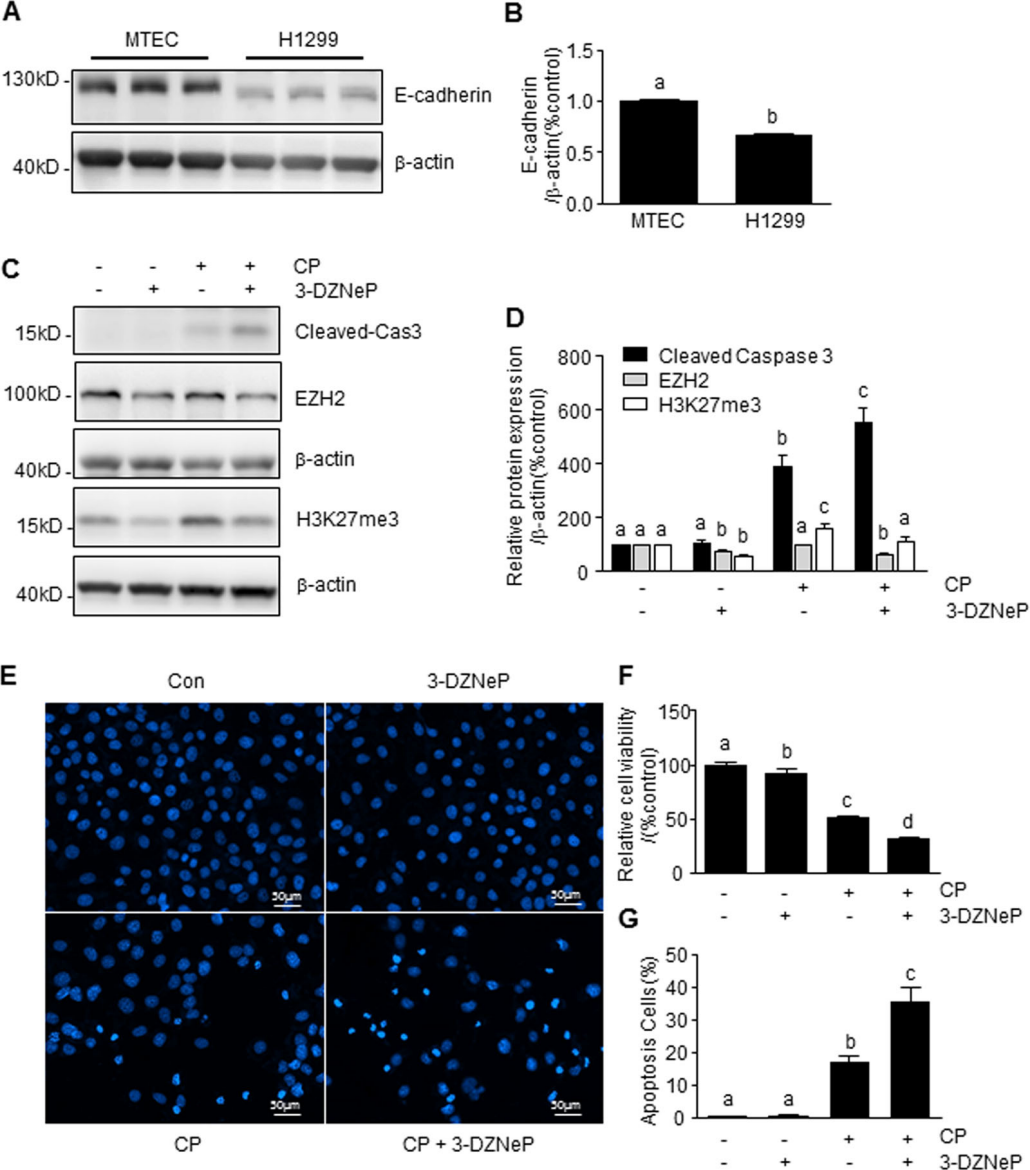
3-DZNeP enhanced CP-induced apoptosis via the inhibition of EZH2/H3K27me3 pathway in non-small cell lung cancer (NSCLC) cells. mTECs and H1299 were normally cultured (**a**) and H1299 cells were pretreated with 3-DZNeP (10 μM) for 30 min and then exposed to cisplatin (20 μg/ml) for an additional 24 h (**c**, **e**, **f**). Cell lysates were prepared and subjected to immunoblot analysis with antibodies against E-cadherin, cas3, EZH2, H3K27me3, and β-actin (**a**, **c**). The levels of E-cadherin (**b**), cleaved-cas3 (**d**), H3K27me3 (**d**), or EZH2 (**d**) were quantified by densitometry and normalized with β-actin. Cell viability was detected by CCK-8 assay (**f**). Cell apoptosis was detected by DAPI staining and the incidence of apoptosis was analyzed by enumerating nuclear of deep dyeing cells with condensed chromatin (**e**), and expressed as the percentage of apoptotic cells (**g**). Data represent the mean ± SEM of at least three experiments. Bars with different letters (**a**–**d**) for each molecule are significantly different from one another (*P* < 0.05)

A previous study demonstrated that 3-DZNeP increased the sensitivity of A549 cells, a NSCLC cell line, to cisplatin^34^. Here, we utilized H1299 cells, another NSCLC cell line, to clarify the mechanism of action of 3-DZNeP in cisplatin-based chemotherapy of NSCLC. As shown in Fig. 7, the basal level of E-cadherin protein was much lower in H1299 cells compared with mTECs (Fig. 7a, b). Pretreatment with 3-DZNeP reduced EZH2 protein expression both in the absence and presence of cisplatin and resulted in the inhibition of cisplatin-induced H3K27 trimethylation (Fig. 7c, d). This was accompanied by enhanced caspase3-cleavage (Fig. 7c, d), loss of cell viability (Fig. 7f) and apoptosis (Fig. 7e, g). These results are similar to what we have observed in renal epithelial cells with E-cadherin depletion with siRNA and further suggest the importance of sufficient E-cadherin expression in mediating renoprotection of 3-DZNeP against cisplatin-elicited cell killing.

### 3-DZNeP restores loss of E-cadherin and protects against cisplatin-induced AKI

**Fig. 8:**
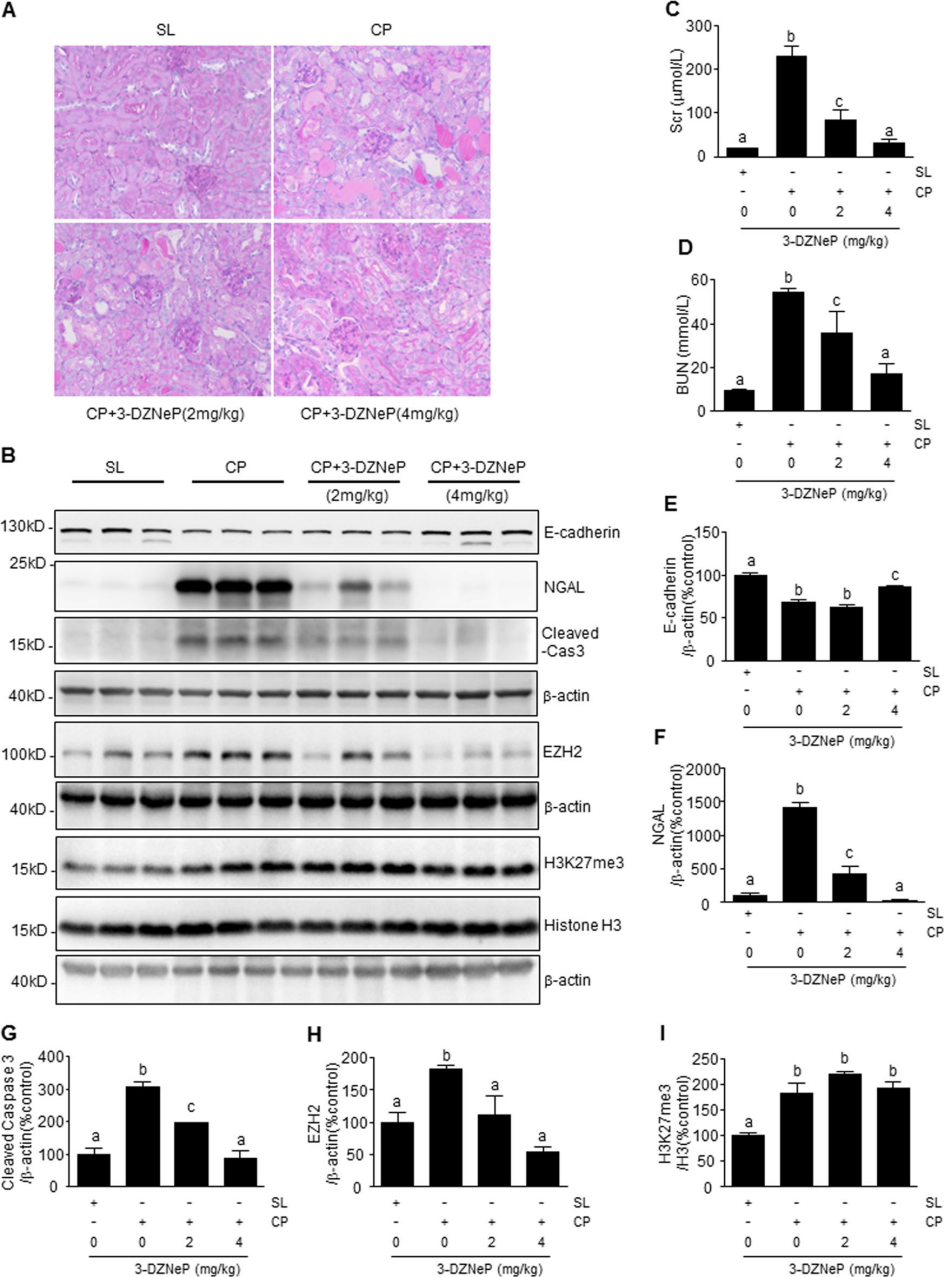
Administration of 3-DZNeP restored loss of E-cardherin and protects against cisplatin-induced acute kidney injury. Representative sections of periodic acid-Schiff (PAS) staining of kidney tissue (magnification ×200) (**a**). Blood was collected at 72 h after cisplatin injection in C57BL/6J mice. Serum creatinine (Scr) and blood urea nitrogen (BUN) were detected as levels of kidney function (**c**, **d**). Kidney tissue lysates were subjected to immunoblot analysis with antibodies against E-cadherin, neutrophil gelatinase-associated lipocalin (NGAL), caspase-3 (cas3), EZH2, H3K27me3, H3, and β-actin (**b**). Data represent the mean ± SEM of at least three mice. The levels of E-cadherin (**e**), NGAL (**f**), cleaved-cas3 (**g**), EZH2 (**h**), or H3K27me3 (**i**), were quantified by densitometry and normalized with β-actin or H3 as indicated. Data represent the mean ± SEM of at least three mice. Bars with different letters (**a**–**c**) for each molecule are significantly different from one another (*P* < 0.05). SL saline, CP cisplatin

To evaluate the therapeutic effect of 3-DZNeP in vivo, we utilized a murine model of AKI induced by cisplatin. At day 3 after receiving cisplatin injection (25 mg/kg), mice developed kidney injury characterized by damage to renal tubular cells as indicated by periodic acid Schiff (PAS) staining, increased expression of neutrophil gelatinase-associated lipocalin (NGAL), an early renal injury biomarker, and increased caspase3-cleavage (Fig. 8a, b). The mice also demonstrated renal dysfunction as evidenced by increases in serum creatinine (Scr) and blood urea nitrogen (BUN) (Fig. 8c, d). Coincident with those changes, E-cadherin expression was significantly reduced in the injured kidneys of the animals (Fig. 8b). Treatment with 3-DZNeP dose-dependently attenuated histologic markers of injury and improved renal function. At 4 mg/kg, 3-DZNeP returned Scr and BUN to the basal levels and reduced renal tubular damage in mice given cisplatin (Fig. 8a–d). Furthermore, 3-DZNeP administered at 4 mg/kg largely restored E-cadherin levels (Fig. 8b, e) and repressed NGAL expression (Fig. 8b, f) and caspase 3 cleavage (Fig. 8b, g). Cisplatin administration increased expression levels of both EZH2 and H3K27me3. 3-DZNeP treatment dose-dependently inhibited EZH2 expression, but did not affect H3K27me3 protein levels. Expression levels of histone H3 were the same in all the groups (Fig. 8b, h, i). These data suggest that protection against AKI conferred by 3-DZNeP is strongly associated with preservation of E-cadherin expression in the cisplatin-damaged kidney.

## Discussion

Cisplatin-induced nephrotoxicity and tumor resistance to cisplatin as chemotherapy remain clinical problems. In this study, we observed that in renal tubular cells, treatment with 3-DZNeP protected against cisplatin-induced tubular cell apoptosis, as evidenced by ameliorated caspase3 activation, improved cell viability, reduced DNA chromatin condensation, and nuclei degradation in vitro, as well as improved renal function and attenuated histopathological changes in vivo. On the contrary, in NSCLC cells, 3-DZNeP treatment increased cisplatin sensitivity as demonstrated by enhanced cytotoxic effects. These data suggest that combined administration of 3-DZNeP and cisplatin would potentiate the anti-tumor effect of cisplatin but reduce its nephrotoxicity.

Although cisplatin is effective in treating various types of solid tumors, its application is limited by tumor resistance and side effects, including nephrotoxicity^5–8^. We observed the cytotoxic effect of cisplatin in both cultured mTECs and tumor cells^12,36^. Interestingly, treatment with 3-DZNeP repressed cisplatin-induced cytotoxic effects in renal epithelial cells while enhancing its ability to kill tumor cells in NSCLC. Initially, we thought that 3-DZNeP protected renal tubular epithelial cells through its effects on EZH2, since EZH2 is the major target of 3-DZNeP and treatment with 3-DZNeP can induce EZH2 degradation and subsequent demethylation of H3K27me3^20^. Surprisingly, our results showed that addition of cisplatin to cultured mTECs did not increase EZH2 expression though it induced H3K27 trimethylation, while 3-DZNeP at doses that attenuated cisplatin-induced renal tubular cell death did not affect the expression level of EZH2 or H3K27me3. These data suggest that protection of renal epithelial cells treated with 3-DZNeP and exposed to cisplatin may not be through EZH2 and H3K27me3, and that cisplatin-induced H3K27 trimethylation is not subject to the regulation of EZH2 in vitro. This is supported by data from our animal studies showing that 3-DZNeP treatment did not inhibit a cisplatin-induced increase of H3K27 trimethylation in the kidney of mice. However, administration of cisplatin to mice resulted in upregulation of EZH2 in the kidney, which was abolished by 3-DZNeP, unlike the effect of 3-DZNeP on EZH2 in cultured mTECs exposed to cisplatin. Currently, the reason for the difference between in vitro and in vivo EZH2 expression in response to cisplatin and the effect of 3-DZNeP is not clear. Studies have shown that in addition to tubular cells, EZH2 is expressed in other cell types, such as interstitial fibroblasts^37^, podocytes^38^, and mesangial cells^39^ in the kidney. EZH2 has also been reported to be expressed in inflammatory cells, such as macrophages^40^, which are usually recruited to the kidney following the injury. It is possible that EZH2 expression in these cell types is increased in response to cisplatin and more sensitive to 3-DZNeP treatment. Further investigation is needed to address these issues.

It appears that a basal level of EZH2 is essential for protecting against cisplatin-induced renal tubular cell damage. This is evidenced by our observations that although cisplatin treatment did not alter the expression level of EZH2, silencing of EZH2 with its specific siRNA still enhanced caspase-3 cleavage and apoptosis in cultured mTECs. This result is opposite to what we have observed in renal epithelial cells exposed to oxidative stress, in which siRNA-mediated knockdown of EZH2 reduced apoptosis. The reasons for the discrepancy in the role of EZH2 in response to different stimuli are currently unclear in vitro system. Our recent studies showed that exposure of renal epithelial cells to oxidative stress (H_2_O_2_) resulted in a remarkable elevation of EZH2^19^, which is different from cells exposed to cisplatin in which there is no change in EZH2 level. Therefore, it seems that a basal level of EZH2 is required for cell survival whereas its upregulation in response to an insult (i.e. oxidative stress) contributes to cell death; overexpressed EZH2 in renal epithelial cells may trigger a cellular mechanism that overrides the survival benefit of basal EZH2, leading to cell death. A similar phenomenon has been observed in other studies. For example, a slight and transient activation of EGFR is required for regenerative response whereas enhanced and prolonged activation of EGFR leads to renal fibrosis^41^.

To identify the mechanism by which 3-DZNeP protects against cisplatin nephrotoxicity, we investigated the effect of 3-DZNeP on the activation of p38, JNK1/2, and ERK1/2, three major intracellular pathways that contribute to cisplatin nephrotoxicity^14–16^, and expression of E-cadherin, a major adhesion molecule of tubular epithelial cells that plays protective role in cisplatin-induced tubular cell apoptosis^35^. Our data showed that 3-DZNeP treatment did not affect cisplatin-induced p38, JNK1/2, and ERK1/2 phosphorylation, but restored the loss of E-cadherin in cultured renal tubular cells treated with cisplatin. Additionally, knockdown of tubular E-cadherin reversed the renoprotective effect of 3-DZNeP, leading to enhanced apoptosis of renal cells following cisplatin exposure. These data suggest that preservation of E-cadherin expression is critical for the cytoprotective effect of 3-DZNeP. This observation is also supported by our findings that 3-DZNeP treatment resulted in E-cadherin preservation in the kidney of mice exposed to cisplatin, whereas, in H1299 cells, a NSCLC cell line with low expression of E-cadherin, 3-DZNeP application potentiates rather than ameliorates cisplatin-induced tumor cells killing. E-cadherin-mediated cell–cell adhesion has been shown to be critical for activation of PI3 kinase/AKT, a well-recognized survival signaling pathway in renal epithelial cells^18^. Thus variable activation of the PI3K/Akt signaling pathway may at least in part account for 3-DZNeP’s protection of renal tubular cells against cisplatin toxicity while enhancing the sensitivity of cancer cells to cisplatin.

Currently, it remains unclear how 3-DZNeP treatment leads to E-cadherin preservation in renal epithelial cells. E-cadherin is a well-known Ca^2+^-dependent transmembrane receptor that is subject to repression by multiple mechanisms including transcriptional repression. The transcriptional factor Snail1 is a repressor of E-cadherin (CDH1) gene expression that acts by binding directly to the E-cadherin promotor^42^. This process requires EZH2-mediated trimethylation of H3K27^42^. However, cisplatin-induced trimethylation of H3K27 was not inhibited by 3-DZNeP in vitro and in vivo, suggesting that 3-DZNeP may preserve E-cadherin expression through a mechanism independent of EZH2-induced trimethylation of H3K27 in epithelial cells. Nonetheless, we cannot rule out the possibility that 3-DZNeP suppresses other repressive markers and associated methyltransferases or other mechanisms that are essential for E-cadherin expression. In this regard, a recent report indicated that 3-DZNeP is capable of inhibiting trimethylation of H3K9, another marker associated with transcriptional repression of E-cadherin^43^. Given that H3K9 methylation relies mostly on Suv39H1, one of the H3K9 methyltransferases^44^, it would be interesting to examine whether Suv39H1 or other methyltransferases that regulate H3K9 methylation contribute to the preservation of E-cadherin expression by 3-DZNeP treatment in renal cells.

In addition, 3-DZNeP may ameliorate renal tubular cell injury by reducing generation of endogenous adenosine and homocysteine through inhibition of S-adenosyl homocysteine hydrolase (SAHH), an enzyme of the activated methyl cycle that catalyzes the hydrolysis of S-adenosyl-l-homocysteine to adenosine and homocysteine^45^. Application of 3-DZNeP, a potent inhibitor of SAHH, not only results in S-adenosyl-l-homocysteine accumulation in cells, but subsequently, inhibits the activities of histone methyltransferases, such as EZH2 while also reducing adenosine and homocysteine levels^46^. It has been reported that hyperhomocysteinemia can enhance cisplatin toxicity in renal tubular epithelial cells by inducing endoplasmic reticulum stress and decreasing AKT activity^47^; elevated adenosine is also involved in cisplatin-induced nephrotoxicity by increasing renal vasoconstriction and thereby decreasing glomerular filtration^48^. The decline in homocysteine and adenosine resulting from SAHH inhibition may also contribute to a 3-DZNeP-mediated renoprotective effect by reducing the susceptibility of renal cells to cisplatin and improving blood supply to the kidney. This hypothesis is worthy of being tested.

3-DZNeP effectively prevents tumor progression by inducing apoptosis as well as inhibiting proliferation^20,49^. In addition, 3-DZNeP has been shown to reduce cancer cell migration and invasion by reversing epithelial-to-mesenchymal transition (EMT), which is characterized by the up-regulation of epithelial phenotype genes, such as E-cadherin^50^. The present study revealed that E-cadherin expression is less in tumor cells than in mTECs. As EZH2 and H3K27me3 expressions lead to transcriptional repression of tumor suppressor genes, including E-cadherin, and promote tumor progression^25,26^, it is anticipated that inhibition of EZH2 and H3K27me3 would enhance the sensitivity of tumor cells to cisplatin. Indeed, our data showed that treatment with 3-DZNeP potentiated the pro-apoptotic effect of cisplatin in tumor cells (H1299) that have a low level of E-cadherin. Since 3-DZNeP exerts a protective effect in the renal tubular cells and a detrimental effect in tumor cells, combined administration of 3-DZNeP and cisplatin in an animal model with solid tumors could demonstrate a role for joint administration of 3-DZNeP and cisplatin as a way to kill tumor cells while reducing harm to the kidneys.

In summary, the results of this study demonstrate for the first time that 3-DZNeP can protect against cisplatin nephrotoxicity and increase sensitivity of tumor cells to this compound. The beneficial effect of 3-DZNeP is associated with preservation of E-cadherin expression. As E-cadherin expression is essential for protection against various insults and prevention of tumor cell migration and invasion^51,52^, combined application of 3-DZNeP and cisplatin might provide a novel chemotherapeutic strategy for treating patients with solid tumors.

## Materials and methods

### Antibodies and reagents

3-DZNeP was purchased from Selleckchem (Houston, TX, USA). Cisplatin was purchased from Sigma (St. Louis, MO, USA). Antibodies to caspase3, E-cadherin, EZH2, H3K27me3, p-JNK, p-ERK1/2, p-p38, histone H3, and tubulin were purchased from Cell Signaling Technology (Danvers, MA, USA). Antibody to NGAL was purchased from R&D systems (Minneapolis, MN, USA). Antibodies to GAPDH, β-actin, horseradish peroxidase-conjugated secondary antibodies, enhanced chemiluminescence (ECL) detection kit, cell counting kit-8 (CCK-8), and DAPI were from Beyotime institute of Biotechnology (Jiangsu, China). Scr and BUN reagent kits were purchased from Nanjing Jiancheng Bioengineering Institute (Nanjing, China). EZH2 siRNA, E-cadherin siRNA and scramble siRNA were purchased from Santa Cruz Biotechnology (Santa Cruz, CA, USA).

### Cell culture and treatment

Mouse kidney proximal tubular epithelial cell line (a gift from Dr. Jeffrey B. Kopp, National Institutes of Health)^53^ was cultured in DMEM with F12 containing 10% FBS. The human NSCLC cell line (H1299) was obtained from the Cell Bank of the Chinese Academy of Sciences (Shanghai, China) and cultured in RPMI-1640 medium containing 10% FBS. Cells were kept in an atmosphere of 5% CO_2_ at 37 °C. Before the experiment, cells were starved with medium containing 0.5% FBS for 24 h to obtain quiescent cells. Cells were treated with cisplatin at different concentrations for a designated time period before harvesting. To determine the effect of 3-DZNeP on cisplatin-induced cell apoptosis, cells were pretreated with diluent (distilled water) or 3-DZNeP at different concentrations for 30 min and then, exposed to cisplatin (20 μg/ml) for 24 h.

### Transfection of siRNA

The siRNA oligonucleotides specifically targeted mouse EZH2 (Santa Cruz Biotechnology, Santa Cruz, CA, USA) or E-cadherin (Santa Cruz Biotechnology, Santa Cruz, CA, USA) were used to down-regulate EZH2 or E-cadherin. MTECs were seeded in antibiotic-free medium and grown for 24 h, then transfected with siRNA (25 nM) specific for EZH2 or E-cadherin facilitated by Lipo-fectamine 2000 Reagent (Invitrogen-Thermo Fisher Scientific, Carlsbad, CA, USA) according to manufacturer’s instructions. In parallel, scrambled siRNA (25 nM) was used as a negative control. 24 or 48 h after transfection, cells were subjected to diluent (distilled water) or 3-DZNeP (10 μM) pretreatment for 30 min when necessary and then exposed to cisplatin (20 μg/ml) for 24 h.

### CCK-8 assay

Cell viability was determined using CCK-8 assay according to the manufacturer’s instructions. Cells were seeded in 96-well plates and then transfected with siRNA and/or pretreated with 3-DZNeP at different concentrations for 30 min and then exposed to cisplatin (20 μg/ml). After treatment for 24 h, cells were exposed to CCK-8 solution for 2 h. Absorbance at 490 nm (OD490) was detected by microplate reader.

### Apoptosis assay

Cell apoptosis was detected by DAPI staining in vitro. Cells grown in six-well plates were transfected with siRNA and then/or subjected to diluent (distilled water) or 3-DZNeP (10 μM) pretreatment for 30 min when necessary and then, exposed to cisplatin (20 μg/ml). After treatment for 24 h, cells were harvested and then fixed with 4% paraformaldehyde, stained with DAPI solution for 15 min and then visualized and photographed under fluorescence microscopy. Apoptotic cells were recognized as punctate nuclear ghosts after DAPI staining. The incidence of apoptosis was analyzed by counting the nuclei of deep dyeing cells with condensed chromatin and calculating the percentage of apoptotic cells.

### Animals and treatment

Male C57BL/6J mice (25–28 g) were obtained from Shanghai SLAC Laboratory Animal Co. Ltd. (Shanghai, China). To establish a mouse model of cisplatin-induced AKI, the animals were injected intraperitoneally with cisplatin (25 mg/kg) in saline. In order to investigate the effect of 3-DZNeP on AKI, 3-DZNeP (2, 4 mg/kg) in distilled water was given intraperitoneally immediately after cisplatin injection and then administered daily. The vehicle group was injected with an equal volume of saline. Mice were euthanized at day 3 after cisplatin injection. Kidney samples were collected for histological examination and the renal cortex was dissected and used for Western blot analyses. Serum was collected for the measurement of Scr and BUN. All the experimental procedures were approved by the Institutional Animal Care and Use Committee at Tongji University, China.

### Renal function analysis

Scr was detected by a Creatinine Assay Kit and BUN was detected by a BUN kit (Nanjing Jiancheng Bioengineering Institute, Nanjing, China), according to the manufacturer’s instructions.

### Renal histology

Kidneys were fixed in 4% paraformaldehyde and embedded in paraffin. Slides of paraffinized tissue sections (4 μm) were deparaffinized, rehydrated, and washed in distilled water. Sections were stained using the PAS method. Histological changes, such as degree of tubular injury were visualized and photographed under microscopy at ×200 optical magnification.

### Western blot analysis

Cell lysates and renal cortex homogenates were prepared for Western blot analysis as previously described^54^. The signals were then imaged by chemiluminescence for 0.1–1 min to visualize signals and quantified using the ImageJ program (http://imagej.nih.gov/ij/). The ratio of the protein examined was normalized against GAPDH, tubulin, β-actin, or histone H3 signals with relative protein levels expressed as fold-induction over controls and expressed as mean ± standard error of the mean (SEM).

### Statistical analysis

Data obtained from this study were expressed as mean ± SEM. Statistical analyses were performed using one-way ANOVA, followed by a Newman–Keuls posttest. The differences between two groups were compared by Student’s *t*-test using Prism 5.0 (GraphPad Software, San Diego, CA, USA). *P* < 0.05 was considered as statistically significant difference between mean values.
