# Correction: 3-deazaneplanocin A protects against cisplatin-induced renal tubular cell apoptosis and acute kidney injury by restoration of E-cadherin expression

**DOI:** 10.1038/s41419-019-1725-8

**Published:** 2019-07-18

**Authors:** Jun Ni, Xiying Hou, Xueqiao Wang, Yinfeng Shi, Liuqing Xu, Xiaoqing Zheng, Na Liu, Andong Qiu, Shougang Zhuang

**Affiliations:** 1 Department of Nephrology, Shanghai East Hospital, Tongji University School of Medicine, Shanghai, China; 2 Shanghai Institute of Immunology, Department of Immunology and Microbiology, Shanghai Jiao Tong University School of Medicine, Shanghai, China; 3 School of Life Sciences and Technology, Tongji University, Shanghai, China; 4 Department of Medicine, Rhode Island Hospital and Brown University School of Medicine, Providence, RI USA

**Keywords:** Pharmacology, Translational research

**Correction to: Cell Death & Disease**

10.1038/s41419-019-1589-y published online 01 May 2019

After publication of this article, the authors found some mistakes in the author information. The correct list of author information should be as following:

Jun Ni^1,2^, Xiying Hou^1^, Xueqiao Wang^3^, Yinfeng Shi^1^, Liuqing Xu^1^, Xiaoqing Zheng^3^, Na Liu^1^, Andong Qiu^3^, Shougang Zhuang^1,4^

^1^Department of Nephrology, Shanghai East Hospital, Tongji University School of Medicine, Shanghai, China

^2^Shanghai Institute of Immunology, Department of Immunology and Microbiology, Shanghai Jiao Tong University School of Medicine, Shanghai, China

^3^School of Life Sciences and Technology, Tongji University, China

^4^Department of Medicine, Rhode Island Hospital and Brown University School of Medicine, Providence, RI, USA

Correspondence: Shougang Zhuang (szhuang@lifespan.org)

^1^Department of Nephrology, Shanghai East Hospital, Tongji University School of Medicine, Shanghai, China

^4^Department of Medicine, Rhode Island Hospital and Brown University School of Medicine, Providence, RI, USA

This has been corrected in both the PDF and HTML versions of the Article.

